# Inspiring Student Entrepreneurship and Innovation

**DOI:** 10.7759/cureus.71195

**Published:** 2024-10-10

**Authors:** Jhillika Patel, Sanjana Eranki, Isabella Tan, Isaac Huang, Trisha Sindhu, Daniel Lam, Rohit Mukherjee, Abhishek Chakraborty, Paul Weber

**Affiliations:** 1 Department of Medicine, Rutgers Robert Wood Johnson Medical School, Piscataway, USA; 2 Department of Continuing Medical Education, Rutgers Robert Wood Johnson Medical School, Piscataway, USA

**Keywords:** diversity and inclusion, medical education, medical entrepreuneurship, medical innovation, student programming

## Abstract

Background

While student entrepreneurial interest is significantly correlated with the intention to pursue innovative healthcare ventures, many medical schools lack formal programs to nurture entrepreneurial skills. To investigate student interest in entrepreneurship and their experiences with medical innovation, the Biomedical Entrepreneurship Network at Rutgers Robert Wood Johnson Medical School engaged students nationally in a Healthcare Innovation Summit*,* featuring a keynote address, pitch competition, and interactive workshops with accomplished innovators.

Methods

A Rutgers electronic Institutional Review Board (eIRB)-approved survey was disseminated to student attendees at the summit, with questions categorized into four themes: interest/motivation, barriers to ideation and execution, support for innovation, and confidence in entrepreneurial pursuits. Responses were quantified using a Likert scale, and qualitative feedback was thematically analyzed.

Results

Among the 54 respondents, 100% exhibited a significant interest in healthcare entrepreneurship. Students cited the following barriers: inaccessible resources (44%), insufficient funding (51%), lack of mentorship (60%), and time limitations (82%). The quantitative thematic analyses demonstrated that students desired formal education on pursuing innovation and dedicated assistance to foster critical thinking. While students were confident in their ability to pursue entrepreneurship, they were not confident in the necessary details required for successful endeavors and avenues for mentorship.

Conclusions

Pursuing healthcare entrepreneurship presents numerous barriers for medical students. Integrating formal entrepreneurship education - emphasizing mentorship and connection between medical students and postgraduate trainees, funding, and critical innovative thinking - into medical curricula can empower students in the pursuit of innovation-based projects and positive change throughout their careers, enhancing access, equity, and quality of healthcare.

## Introduction

Medicine and innovation are becoming increasingly intertwined as healthcare technology rapidly evolves, expanding access and new applications to diagnostics and treatment. As shown by Watson et al., student entrepreneurial interest is significantly and positively correlated with the intention to pursue innovative healthcare ventures [1]. Though there has been an increase in innovation programs among medical school curricula in the United States, they are unstandardized and vary in concentration. A majority of these innovation-based programs aimed at the medical student level emphasize law/regulation, business, design, and prototyping as part of a capstone project for evaluation [2]. These programs differ greatly depending on available resources, funding, institutional policies, and level of integration within the core curriculum [3]. The specific goals of each program are also highly variable despite similar focuses on teaching objectives, as core competencies are designed by individual program directors. Primarily, innovation-based programs in an academic setting are presented to students as a separate focus area or optional capstone work or case studies that are not a required portion of the preclerkship or clerkship curriculum [4,5].

A paucity of medical schools provides formal curricular opportunities to cultivate student entrepreneurial skills that can be translatable to their post-graduate training. As shown by Watson et al., student entrepreneurial interest was significantly and positively correlated to the pursuit of innovative healthcare ventures. Curriculum-based efforts that foster interest can bolster confidence, interest, and intent, ultimately inspiring students to pursue innovative healthcare ventures. This research seeks to expand on the prior study by assessing students' entrepreneurial interests and perceived barriers to involvement. To discern the barriers students experience when pursuing entrepreneurial ideas, the Biomedical Entrepreneurship Network, a sanctioned student interest group at Rutgers Robert Wood Johnson Medical School (RWJMS), engaged students nationwide in a Healthcare Innovation Summit. Events included a keynote address, a pitch competition with expert judges, and open dialogue with accomplished innovators through panels and workshops. Judges and panelists spoke about their experiences integrating innovation and entrepreneurship into their careers in healthcare as well as opportunities that exist for students to become engaged in programs/projects throughout various stages of their careers. The event culminated with a pitch competition wherein medical students from across the country pitched ideas for medical devices, mobile applications, and community health programs. Student teams were given feedback from judges with clinical and innovation experience, giving them insight into various dimensions of medical technology creation, development, and usage.

This study seeks to elucidate student interest in innovation and entrepreneurship, recognize limitations in the current programs that are available to them, and gain a greater understanding of how to facilitate student involvement in these programs. The specific hypothesis is that medical students are interested in student entrepreneurship and desire formal skill-based learning.

## Materials and methods

Research design and ethical approval

The research adopted a mixed-methods approach to comprehensively examine students' interests and perceived barriers to engagement in healthcare innovation post-participation in a virtual Health Innovation Summit. Ethical approval for the study was secured from the Rutgers Institutional Review Board (IRB) (IRB Protocol Number 2022002082).

Data collection instrument and participant engagement

An 18-item IRB-approved survey was the principal instrument for data collection, distributed among student attendees after the Health Innovation Summit. The surveys were sent by email by the Biomedical Entrepreneurship Network (BEN) leadership team at RWJMS. This instrument covered various categories, including demographics, motivation, barriers to idea development, career aims, diversity, and support/networking. Participation required informed consent. Participants completed the survey after attending all components of the Health Innovation Summit, comprising a keynote address, a pitch competition, and open dialogues with expert panels and workshops. The total number of participants was 76 students. The summit was conducted virtually. A web-based survey was administered to all participants who met the inclusion criteria, with data collection taking place over a one-week period following the conclusion of the summit. To be included, participants were required to complete all summit sessions and be current students. Exclusion criteria included individuals not enrolled as students or those who did not attend the entire summit. A reminder was sent midway through the data collection period. Participants were allowed to skip any questions they were uncomfortable answering, yielding a variety in total respondents for certain questions.

Thematic analysis

Thematic analysis was employed to identify qualitative insights from responses to three qualitative free-response questions. Each question was crafted to target specific dimensions of participants' perspectives on healthcare innovation, providing a holistic exploration of qualitative data. The methodological approach to thematic analysis involved a process of code development. Codes were systematically generated to capture recurrent ideas, sentiments, or topics emerging from participants' responses to the survey question and grouped to inform the comprehensive analysis.

Weighting analysis

A cell-based weighting analysis was conducted specifically for question 9, in which participants were asked to explain their ranking for the importance of conference organizers endorsing speakers who are diverse in their backgrounds and experiences. The purpose was to correct potential imbalances in the sample composition. By employing a cell-based weighting approach, the analysis will account for variations in the sample composition, particularly focusing on demographic factors such as age, gender, race, and ethnicity. This method assigned values to responses on a Likert scale. This scale allowed for a nuanced assessment of the importance participants placed on organizers choosing diverse speakers.

Quantitative data analysis

Quantitative data analysis involved mean score computation, standard deviations, and variances for questions related to confidence levels and motivation. Statistical tests, including ANOVA and t-tests, were employed to discern significant differences between groups. Further analyses included regression analysis, Pearson correlation coefficient (r), and comparative analyses, which explored relationships, correlations, and variations across different variables and demographic groups.

## Results

Demographics

From the survey results and data analysis, the first set of questions provided a general characterization of the respondent demographics. Fifty-four participants, with the majority (80%, 43/54) falling between the ages of 21 and 30, took part in the Health Innovation Summit and completed the post-event evaluation. Of the respondents, males and females were equally represented. Additionally, the majority of survey participants (63%, 34/54) identified as medical students, with the identification of undergraduate (15%, 8/54), master’s (11%, 6/54), and PhD students (6%, 3/54). Asian Americans comprise 50% (27/54) of the sample, followed by White (24%, 13/54), Black or African American (19%, 10/54), Hispanic or Latino (4%, 2/54), and Native Hawaiian or Pacific Islander (2%, 1/54) (Table 1).

**Table 1:** Participant demographics

Demographic category	Subcategory	Number of participants	Percentage (rounded to nearest whole number)
Age group	<21	3	6%
	21-25	27	50%
	26-30	16	30%
	31+	4	7%
	Declined to answer	4	7%
Gender	Male	24	44%
	Female	24	44%
	Other	2	4%
	Declined to answer	4	7%
Ethnicity	Asian American	27	50%
	Black or African American	9	17%
	Declined to answer	2	4%
	Hispanic or Latino	2	4%
	Native Hawaiian or Pacific Islander	1	2%
	White	13	24%

Motivation and interest

Participants’ motivations and interests toward entrepreneurship and innovation were assessed with multiple answer choices allowed. Regarding motivation and interest, 45 out of 54 respondents chose to answer the questions with the ability to check multiple options. When asked about motivations for taking part in the RWJMS Healthcare Innovation Summit, 84% (38/45) of respondents indicated they desired to learn more about healthcare innovation (Table 2). With regards to hearing directly from a speaker about entrepreneurial experiences, 71% (32/45) indicated interest. Students were also motivated to gain opportunities for mentorship (56%, 25/45), hear student teams pitch ideas with expert feedback (38%, 17/45), and participate in a question-and-answer session with panelist speakers (22%, 10/45). When asked to identify aspects of entrepreneurship that interested them the most, participants indicated working to solve problems, being in spaces designed for innovative thinking, the scale of potential impact, networking opportunities, and pitching ideas. In terms of expected support from their communities, participants thought overall they would be met with approval, but with slight differences among different groups. On a Likert scale assessing approval of their communities for participation in entrepreneurship ranked from 1 (representing no approval) to 5 (representing full approval), participants rated a mean score of approval of 4.31 among close family, 4.21 among friends, 3.95 among colleagues, and 4.10 among mentors. The statistically significant lower score for expected support among colleagues (p < 0.05) may allude to attitudes, real or perceived, of entrepreneurship in the workplace in general.

**Table 2:** Motivation toward pursuing entrepreneurship and innovation

Motivators	# participants endorsing
To learn more about healthcare innovation	38/45 (84%)
To hear directly from a speaker about healthcare innovation experiences	32/45 (71%)
Opportunity for mentorship throughout career	25/45 (56%)
Hearing other teams pitch their ideas	17/45 (38%)
Participation in a Q&A panel with panel speakers	10/45 (22%)
Other	3/45 (7%)

Barriers to pursuing entrepreneurial endeavors

When asked about barriers and concerns to pursuing entrepreneurial endeavors as future healthcare professionals, 82% (37/45) of students cite lack of time, 44% (20/45) cite inaccessible resources, 51% (23/45) cite lack of funding for their ideas, and 60% (27/45) feel as if they have a lack of mentorship/guidance. Thematic analysis was conducted on free-response questions asking students to further describe their barriers, yielding common themes across responses. Overall, students feel that resources at schools are insufficient and inaccessible. Students also cite a lack of available mentors/guidance specifically on getting started with ideation and time as the most prominent barriers. A prominent theme noted was the lack of information and awareness about existing resources. In addition, students noted institutional barriers and suggested in response that curriculums should provide opportunities to think creatively and innovatively about health solutions. Medical students, in particular, note the lack of formal education and resources to pursue their novel ideas within the healthcare space and the desire to have dedicated resources and help with team formation, ideation, market analysis, and next steps for their innovative ideas. Students also expressed that the environment is not encouraging for them to pursue their ideas, and some students articulated they have experienced discouragement, contributing to a lack of confidence. Students stated that they especially need a program to help them with the first few steps of creating their ideas and moving the projects forward, specifically with funding, support, and faculty willingness to support in the beginning stages. Medical students noted the pressure to prioritize schoolwork and research over pursuing entrepreneurial interests.

Participants were also asked about their intent to incorporate entrepreneurial ventures into their future careers. When asked if they agree with this statement on a Likert scale from 1 (not confident at all) to 5 (very confident): “My professional goal is to become an entrepreneur in healthcare” (professional goals), results showed a mean score of 3.60 out of 5. The second prompt stated, “I have the firm intention of starting an entrepreneurial project in healthcare in the future” (projects). Results showed a similar mean score of 3.57. Finally, the third prompt stated, “I will incorporate healthcare innovation/health technologies/entrepreneurship in my future career” (innovations). Respondents gave a slightly higher mean score of 4.02 out of 5.

A one-way ANOVA found statistically significant differences in means across the three sentiments across participants (p < 0.0001), and t-tests were used to describe differences between mean confidences. Participants in the survey felt least certain about their intention to start an entrepreneurial project in healthcare (p = 0.05). On the other hand, respondents felt more confident about their professional goals to become entrepreneurs in healthcare (p = 0.015) and most confident about having the intention of incorporating healthcare innovation into their future careers (p = 0.0004).

Initial exposures to entrepreneurship and innovation

A thematic analysis was also performed for short-answer questions about the participant’s first exposure to innovation and entrepreneurship in healthcare. About 40 participants chose to answer questions about their first exposure to innovation and entrepreneurship. The most prominently identified themes were academic sources, comprising 58% (23/40) of responses, which included undergraduate experience, gap year experience, as well as high school and post-graduate experience (Table 3). Other popular themes included personal interest, peer and mentor influence, as well as innovation groups and events. However, from this analysis, many participants reported being first exposed to these topics and themes during their academic careers, 48% (11/23) of which reported first exposure in their undergraduate career.

**Table 3:** Modes of initial exposures to entrepreneurship and innovation

Exposure setting	# participants
Academic (high school, college, medical school)	23/40 (58%)
Other (outside of traditional academic exposure)	17/40 (43%)

Diversity in mentorship and innovation exposure experiences

Diversity is also an important characteristic that the survey investigated. A cell-based weighting analysis was conducted, employing post-stratification to balance the sample. Forty-five respondents chose to answer this question regarding diversity, and 44 indicated that diversity is important to them in some capacity. Participants evaluated the relative importance of organizers in choosing speakers of diverse backgrounds and experiences using a Likert scale. The responses showed that 98% (44/45) of respondents felt it was important to them in some capacity, with 38% (17/45) reporting very important and 42% (19/45) extremely important (Table 4).

**Table 4:** Participants subjective beliefs on importance of diversity in mentorship and innovation exposure experiences

Subjective importance rating	# participants
Not at all important	0/45 (0%)
Slightly important	1/45 (2%)
Moderately important	8/45 (18%)
Very important	17/45 (38%)
Extremely important	19/45 (42%)

Additionally, a thematic analysis was conducted to examine the importance of diversity in entrepreneurial environments. Out of the 45 respondents to this question, 33 respondents opted to provide a short answer to the importance of diversity. After reviewing 33 respondent scripts, four key themes were identified, namely application of interdisciplinary perspectives (expertise in area of focus, personal experience), advancing career development (mentorship and networking, similarity with speakers), inclusion of diverse interests and demographics (coverage of interest, representation), and encouraging entrepreneurship (promoting innovation, novelty). Certain words were revised and reorganized to integrate them cohesively into distinct themes. About 85% (28/33) mentioned a theme within the application of interdisciplinary perspectives, 70% (23/33) mentioned the inclusion of diverse interests and demographics, and 42% (14/33) mentioned both “advancing career development” and “encouraging entrepreneurship.”

Students greatly valued the personal life experiences of the healthcare innovation panel for “highlighting multiple pathways toward innovation in healthcare” and “embolden(ing) participants from different backgrounds to pursue entrepreneurship.” Moreover, respondents additionally valued the individual perspectives of experts in various disciplines. They believed they could apply unique perspectives to engender critical thinking, highlighted by one participant stating, “Most ideas I’ve had in the clinic haven’t directly been related to minority health. So I am mostly looking for financial guidance in large hospital purchasing environments.” Last, students equally value hosting a panel with varied experiences and expertise to advance their career development and encourage innovation. For example, participants stated that it “would have been nice to have a day of networking or student industry leader collaboration sessions to solve mock work problems” and that they found “interdisciplinary learning as essential to innovation and design-thinking.”

Confidence

**Figure 1:**
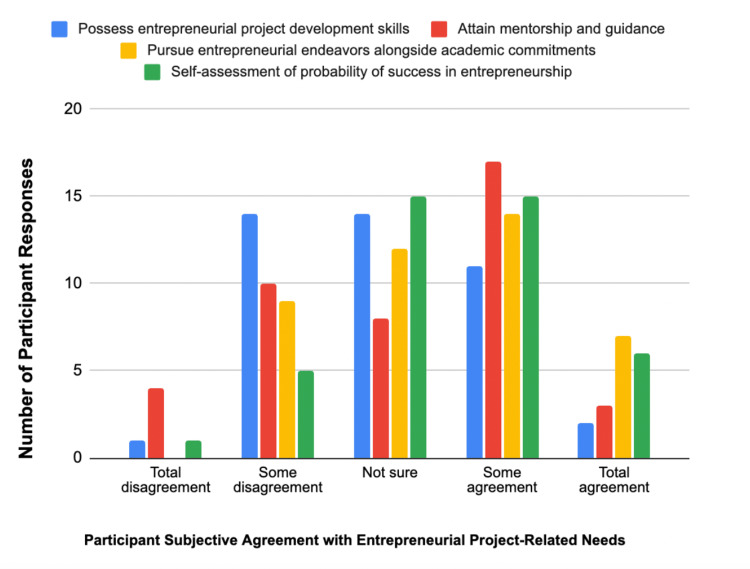
A bar chart displaying the number of participants subjective agreement with each entrepreneurial project-related need

Related questions were designed to explore a participant’s confidence level in their entrepreneurial capacity and direction. Participants were given four statements and asked to what extent they agreed with the sentiments on a scale of 1 (total disagreement) to 5 (total agreement): “I know the necessary details to develop an entrepreneurial project” (knowledge), “I know who to reach out for mentorship” (mentorship), “I can pursue entrepreneurship as a medical student” (capable medical student), and “If I were to pursue entrepreneurship, I would be successful” (success) (Figure 1). It is interesting to note that participants felt that if they were to pursue entrepreneurship/innovation in medicine, they would have a high probability of being successful, with the highest mean score of 3.48. Participants scored the lowest level of agreement with a mean score of 2.98 with the statement that they know the necessary practical details to develop an entrepreneurial project. Single-factor ANOVA did not find statistically significant differences between groups (p = 0.065). However, two-tailed T-tests found a statistically significant difference between participants’ agreement of potential success with their knowledge (p = 0.0075) and capabilities as medical students with their knowledge (p = 0.0034).

Last, confidence in students’ entrepreneurial abilities was assessed following attendance at events at the BEN Healthcare Innovation Summit. Participants were asked, “How confident are you in your entrepreneurial abilities after listening to today’s talks?” on a scale from 1 (not confident at all) to 5 (very confident). The majority of participants indicated they were at least somewhat confident (m = 3.48) in their abilities after the day-long health innovation-based programming, with zero participants indicating no confidence at all.

Deeper relationships

**Figure 2:**
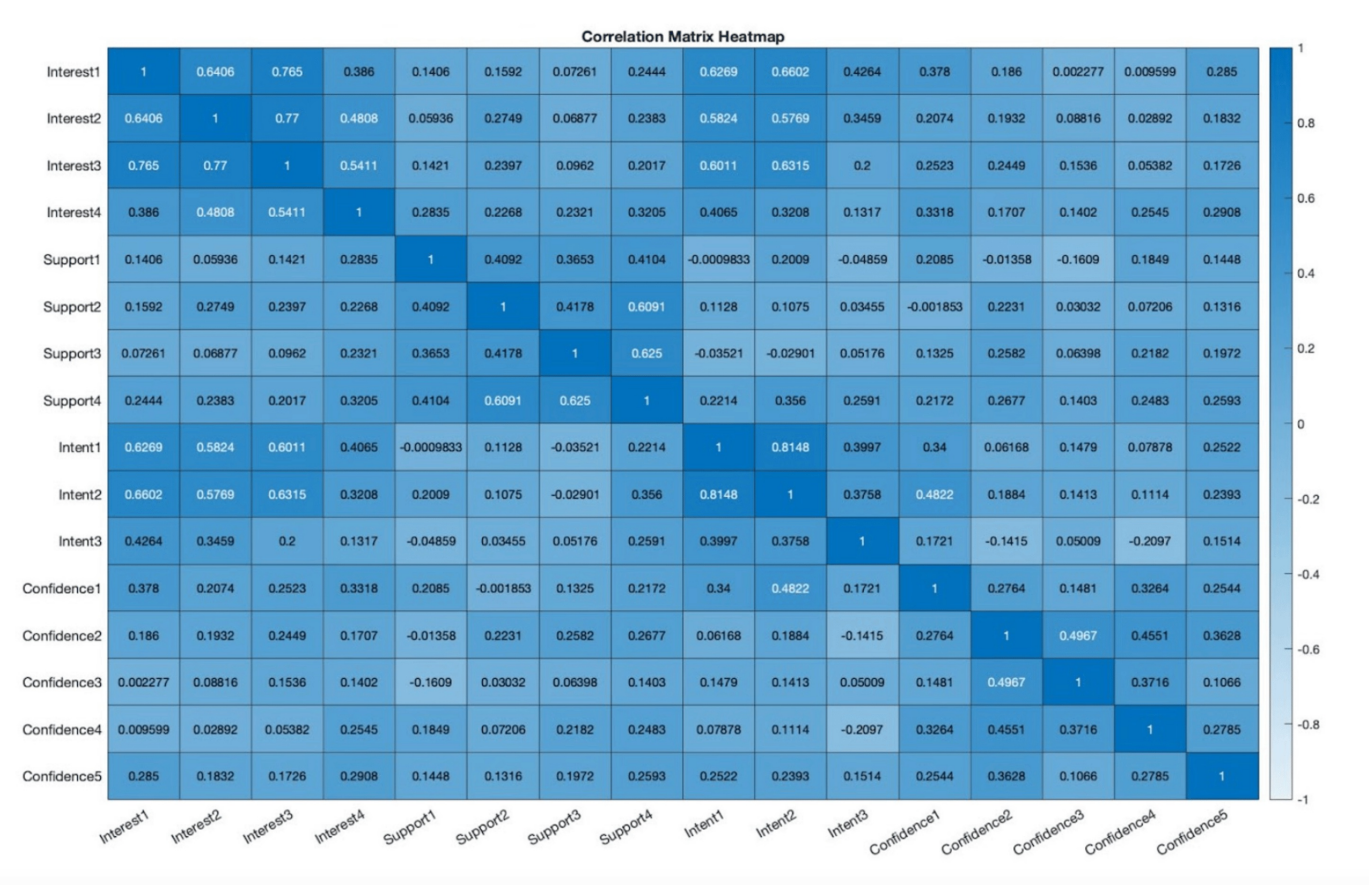
Correlation matrix heatmap displaying the Spearman’s rank correlation between each question pair (interest, support, intent, confidence)

To investigate the overall correlations between interest, support, intent, and confidence of participants, Spearman’s rank correlations were calculated between each question pair (Figure 2).

**Figure 3:**
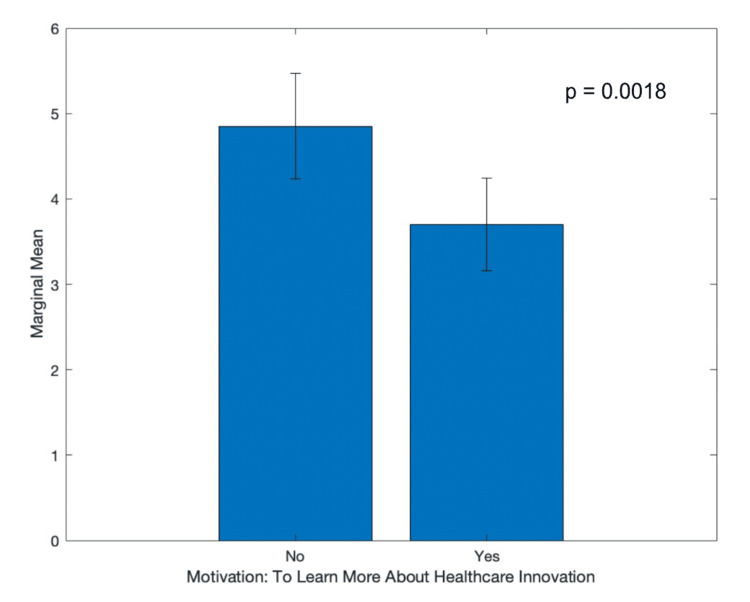
Participant intent to incorporate innovation and entrepreneurship into their future career based on their responses to their motivations

Only the intent 2 variable was able to correlate with confidence (r = 0.48) which stated, “I have the firm intention of starting an entrepreneurial project in healthcare in the future.” Within the confidence questions, confidence 3 (r = 0.50) moderately correlated with confidence 2. The strongest correlation was seen between intent 1 and intent 2 (r = 0.81), which asked about professional goals to become an entrepreneur and the intention of starting a project. Nearly all interest scores were moderate to strongly correlated with each other. There were also moderate correlations seen between participant interest and intent to pursue entrepreneurship (Figure 3). Support notably had moderate correlations between itself (r = 0.61, r = 0.63) but had zero correlations with other categories. 

Additionally, a factorial ANOVA was used to examine the variance contributed to participants’ answers to questions about their confidence and intent. Every “check all that apply” question was broken down into yes or no sub-questions. After accounting for variance across all 17 sub-questions about age, interest, motivations, concerns, and diversity, there were no statistically significant contributions to variance by any of the questions for confidence, though multiple questions involving concerns (p = 0.06) and motivations (p = 0.06) came close. However, when the same ANOVA analysis was applied to intent, participants were significantly more likely to agree with the intent “to incorporate healthcare innovation/health technologies/entrepreneurship in my future career” if they also agreed that they were motivated to attend the summit to “learn more about healthcare innovation” (marginal mean: 4.06 ± 0.6) than if they were not (marginal mean: 2.15 ± 0.5). This question significantly contributed to the variance in intent (F = 12.89, p = 0.0018). This is a very interesting finding that highlights the invaluable experience a summit such as this one provides. Students have shown an enthusiasm to learn about healthcare innovation, especially the ones who plan to incorporate it into their future careers. Higher education institutions then have a responsibility and opportunity to nurture this energy and develop these students to succeed in areas of healthcare innovation, technologies, and entrepreneurship.

## Discussion

The survey results represented a group of participants with a diversity of age ranges, ethnicities, and educational levels. While the ratio of Asian Americans matriculating to medical school (25.67%) is higher than overall American demographics (~7%), self-identified Asians were still overrepresented in this sample at 50% [6]. The analysis of barriers is critical to understanding which roadblocks and initiatives may be worthwhile to target to empower students for entrepreneurial pursuits. Time management was a key issue that may be a perennial barrier to any pursuit for medical students. However, students express interest and motivation to pursue entrepreneurship, while the lack of resources, including funding, mentorship, and formal programs, stunts their ability to do so. These are barriers that academic programs could address if funding and mentorship were held to a higher priority or outlined as a key tenant in schools’ educational goals. These findings suggest that students want to include entrepreneurship and innovation in their careers but may be uncertain about what that entails professionally and how to get started on embarking on projects. Though these challenges to entrepreneurship may not be unique to medical school and higher education, they currently reflect insufficient attention for a group of highly motivated individuals with a rare depth of foundational expertise who are uniquely positioned to find success in these types of endeavors. This data suggests that academic experiences may correlate with exposure to innovation and entrepreneurship in healthcare. It is not a secret that entrepreneurship is a challenging area of study to navigate, and so perhaps future studies should differentiate between ubiquitous challenges unique to medical students and professional students at each stage in their training.

Educational institutions and medical schools should work to establish connections, mentor programs, lecture series, formal education, and other resources to reduce student barriers to exploring and implementing their creative solutions in healthcare. Students will benefit from having an avenue to get resources such as lab and equipment or programming resources to pursue their ideas. Schools such as RWJMS, the University of Texas, Southwestern Medical School, and Carle Illinois College of Medicine are some of the few in the country that have dedicated curricular and school access to these resources. Stronger curriculums need to be made at schools to integrate healthcare innovation. Other ideas that could increase student interest and participation in entrepreneurship include hosting more engagement events around medical innovations, connecting students with physicians who have pursued their ideas in the past, hosting workshops, and connecting students with mentors through a mentorship program.

Diversity of speakers, mentors, and experiences is also an important factor to consider when devising programming and structure for innovation and entrepreneurship at universities. Abstracting from the results of the weighted analysis of 45 response scores to correct for any sampling imbalances in accordance with age, gender, race, and ethnicity, the majority of respondents to the summit survey found that diversity of speaker choice is very important for them. Diversity possesses the synergistic effect of presenting not just different perspectives but also different niche areas of opportunity for innovation that may be hidden otherwise. In addition, diverse speakers provide varied perspectives for students. Diversity of lived and academic experience is particularly critical in fostering open spaces for students to communicate their objectives and concerns while also shedding light on the multiple pathways available in healthcare innovation. The diversity of speakers and mentors also creates greater opportunities for students in terms of networking and guidance for niche pursuits. Pitch competitions, with a diverse panel of judges, are also a unique method for students to attain real-time feedback and meet potential mentors. This is an invaluable opportunity where students learn to represent their product needs and solutions.

Confidence was scored highly across participants, and participants stated they were eager to get involved if given an opportunity. The data demonstrates the need for more resources and development opportunities for formal education and programs dedicated to connecting medical students to the entrepreneurial ecosystem because participants are confident and believe in their success but view lack of knowledge as a ceiling for their confidence. It may also be reasonable to conclude that because of a lack of information, confidence may even be higher if only students were provided with foundation and structure. The lack of information can be categorized into several key areas. Many medical students are unaware of entrepreneurial opportunities, such as internships, startup incubators, or innovation grants, limiting their ability to engage with relevant experiences. Additionally, students often lack awareness of critical resources, including funding mechanisms, legal assistance, and business development tools, which could enhance their confidence in pursuing entrepreneurial ventures. Mentorship is also a crucial factor, yet many students are uncertain about how to identify and connect with experienced mentors, leaving them without essential guidance. Finally, there is a gap in understanding vital processes, such as patent acquisition and regulatory compliance, highlighting the need for structured educational support. It is not the ability that is the issue, but rather basic knowledge. Students felt that the Health Innovation Summit, associated workshops, and panel discussions helped provide some of these requested resources and address some of the challenges present in this space. This may suggest a perceived dichotomy between intrinsic variables (intent, interest, and confidence) and extrinsic variables (support) when it comes to entrepreneurial pursuits. Based on this data and feedback from students, it is clear that programming similar to the Summit where students gain exposure to innovation and entrepreneurship in medicine is beneficial for providing them with resources, mentors, information, and examples of how to integrate innovation into their present or future careers. A general limitation of this study may be the self-selection of participants who were interested in attending a summit dedicated to these topics.

A prior study from 2018 shows that the sampled population of medical students showed positive interest and confidence in pursuing innovation and entrepreneurship [1]. This can be correlated with the findings outlined in this paper. Here, we have further elucidated the barriers and challenges that students perceive in pursuing innovation and entrepreneurship. 

Limitations of this study include that not all participants answered every question, introducing some variability in the sample sizes for each aspect of students’ perspectives on the pursuit of innovation and entrepreneurship. Additionally, this study self-selected for students who had some interest in innovation and entrepreneurship as it was distributed to participants of the summit.

Based on the results of this study, current medical school scholarly project requirements can be expanded to include the identification of one unmet clinical workflow need as witnessed by students and a well-researched potential solution. As an example, scholarly project requirements could be structured at students' respective medical schools or hospital systems, with the identification of one gap or problem in healthcare, brainstorming potential solutions, and constructing an innovation report after choosing one solution. This report can include identifying a problem and researching the gap in the health system, key players/stakeholders, current solutions, an in-depth description of one solution, market analysis, challenges, and next steps. This would influence students to think deeply about their ideas and practice critical thinking in medical innovation. Needs criteria would provide a basis for the creation of an applicable product/workflow and would encourage students to continue to pursue their scholarly ideas under the guidance of clinicians and mentors, who can advise them regarding the feasibility of healthcare workers/patients.

## Conclusions

The results of this study suggest that medical students experience a multitude of obstacles in pursuing innovation-related projects during their education. Students value diversity of demographics and experiences in their mentors and assistance in innovation-based critical thinking. Integrating formal innovation-based modules as relevant to the concurrent medical school curriculum may serve to mitigate these barriers. Creating opportunities for mentorship via direct pairings, networking events, or easy access to a mentorship database may help students feel supported and guided in their pursuits. Further, allocating funding from medical schools and encouraging critical case-based thinking are additional methods of enhancing student exposure to student innovation. These integrations into the curriculum may address the current lack of entrepreneurship in medical education and inspire students to gain insight into the gaps that exist in healthcare, allowing them to improve access, equity, and quality of care. In the future, studies can also be performed examining student feedback on current innovation/entrepreneurship curricula in medical school. By mitigating student barriers to entrepreneurship and innovation, providing opportunities for student-based healthcare innovation, and developing strong mentorship programs, undergraduate medical education can inspire the next generation of health innovation leaders.

## Appendices

Biomedical Entrepreneurial Network (BEN) Healthcare Innovation Summit General Attendee Survey

Start of Block: Demographic Information

This is an 18-question, anonymous survey to be used to share more about your motivation for being part of the Biomedical Entrepreneurial Network (BEN) Healthcare Innovation Summit Day and to learn more about your entrepreneurial interests and future interests. Please fill this out independently.

1. How old are you?

o 18 to 20 years old

o 21 to 25 years old 

o 26 to 30 years old 

o 30+ years old

2. What is your self-identified gender identity?

o Male

o Female 

o Transgender 

o Non-binary / third gender / non-conforming

o Prefer not to say 

3. What is your race/ethnicity?

▢        American Indian or Alaska Native  (1)

▢        Asian  (2)

▢        Black or African American  (3)

▢        Hispanic or Latino  (4)

▢        Native Hawaiian or Other Pacific Islander  (5)

▢        White  (6)

4. What is your current or highest level of education?

o Medical Student  (1)

o Undergraduate Degree  (2)

o Masters Student  (3)

o PhD or Other Doctorate  (4)

o High School  (5)

o Other  (6)

5. After attending the BEN Summit today, what is your stance on incorporating innovation / entrepreneurship / health technologies in your future career?

o Very likely  (1)

o More likely  (2)

o Neutral  (3)

o Less Likely  (4)

o Not Likely  (5)

6. How confident do you feel in your ability to pursue entrepreneurial endeavors as a medical student? On a scale of 1 (Not Confident At All) to 5 (Very Confident in Pursuing Entrepreneurial Endeavors)

1

2

3

4

5

7.  Rate how you feel about this statement between 1 (not at all) to 5 (certain).

If I were to pursue entrepreneurship, I would be successful. 

1

2

3

3

4

5

8. What is your motivation for taking part in the RWJMS BEN Healthcare Innovation Summit? (please check all that apply):

▢        To learn more about healthcare innovation  (1)

▢        To hear directly from a speaker about their entrepreneurial experiences  (2)

▢        Opportunity for mentorship throughout your career  (3)

▢        Hearing other teams pitch their ideas  (4)

▢        Participation in a Question & Answer panel with panelist speakers  (5)

▢        Other  (6)

9. What, if any, are your concerns with pursuing entrepreneurial endeavors as a healthcare professional? (Check all that apply):

▢        Lack of time  (1)

▢        Inaccessible resources  (2)

▢        No funding for ideas  (3)

▢        Lack of mentorship/guidance  (4)

▢        Does not fit into my career goals  (5)

▢        Not interested  (6)

10. In general, what elements of healthcare entrepreneurship interest you the most? (Check all that apply):

▢        Innovative Environment  (1)

▢        Pitching Ideas  (2)

▢        Networking Opportunities  (3)

▢        Scale of Potential Impact  (4)

▢        Working to Solve Problems  (5)

▢        Other  (6)

Display This Question:

If Q10 = Other If you selected "Other" to the previous question, please briefly explain what other elements of healthcare entrepreneurship interest you the most.

________________________________________________________________

11. How important is it to you for Conference Organizers to choose speakers that are diverse in their backgrounds and experiences?

o Not at all important  (1)

o Slightly important  (2)

o Moderately important  (3)

o Very important  (4)

o Extremely important  (5)

Q11b. Please briefly explain your answer choice to the previous question on speaker diversity:

________________________________________________________________

12. Please rate how helpful the panelists today were at helping you understand how healthcare innovation is continually evolving on a scale of 1 (Not helpful) to 5 (Most Helpful)

The panelists today helped me understand how healthcare innovation is continually evolving ()

1

2

3

4

5

13. Indicate your level of agreement from 1 (total disagreement) to 5 (total agreement) for the following statements:

I hope to pursue a career entrepreneurship/innovation as part/or concurrent with my medical career. ()

If I had the opportunity and resources, I'd like to start an entrepreneurial project in healthcare. ()

Being an entrepreneur in healthcare would entail great satisfactions for me. ()

Being an entrepreneur in healthcare implies more advantages than disadvantages to me. ()

1

2

3

4

5

13b. I feel that I would receive an adequate amount of support from my community (friends, family, colleagues, mentors) if I were to pursue an entrepreneurial endeavor in healthcare, on a scale of 1 (not at all) to 5 (absolutely):

My close family would approve of my decision ()

My friends would approve of my decision ()

My colleagues would approve of my decision ()

My mentors would approve of my decision ()

1

2

3

4

5

14. To what extent do you agree with the following statements regarding your entrepreneurial capacity? Indicate your level of agreement from 1 (total disagreement) to 5 (total agreement).

If I were to pursue entrepreneurship/innovation in medicine, I would have a high probability of successful. ()

I know the necessary practical details to develop an entrepreneurial project. ()

I know who to reach out to for mentorship or advice if I want to pursue entrepreneurship/innovation in healthcare. ()

I can pursue entrepreneurial endeavors as a medical student. ()

1

2

3

4

5

14b. Indicate your level of agreement from 1 (total disagreement) to 5 (total agreement).

My professional goal is to become an entrepreneur in healthcare. ()

I have the firm intention of starting an entrepreneurial project in healthcare in the future. ()

I will incorporate healthcare innovation / health technologies / entrepreneurship in my future career. ()

1

2

3

4

5

15. How confident are you in your entrepreneurial abilities after listening to today's talks? On a scale between 1 to 5 (not confident at all to very confident) 

1 

2

3 

4 

5 

16. We are curious if you have access to resources in your home institution to help you with your innovations. Please comment on how you feel the resources you have available to you are. Is there anything you feel that you are lacking in terms of resources or anything that can be stronger?

________________________________________________________________

17. If any, what are the barriers to student entrepreneurship that you have experienced?

________________________________________________________________

18. In general, how and when would you say you were first exposed to innovation/entrepreneurship in healthcare?

________________________________________________________________
